# A meta-data based method for DNA microarray imputation

**DOI:** 10.1186/1471-2105-8-109

**Published:** 2007-03-29

**Authors:** Rebecka Jörnsten, Ming Ouyang, Hui-Yu Wang

**Affiliations:** 1 Department of Statistics, Rutgers, the State University of New Jersey, New Brunswick, NJ 08903, USA; 2 Informatics Institute, University of Medicine and Dentistry of New Jersey, Piscataway, NJ 08854, USA; 3 9 Stoecker Road, Holmdel, NJ 07733, USA

## Abstract

**Background:**

DNA microarray experiments are conducted in logical sets, such as time course profiling after a treatment is applied to the samples, or comparisons of the samples under two or more conditions. Due to cost and design constraints of spotted cDNA microarray experiments, each logical set commonly includes only a small number of replicates per condition. Despite the vast improvement of the microarray technology in recent years, missing values are prevalent. Intuitively, imputation of missing values is best done using many replicates within the same logical set. In practice, there are few replicates and thus reliable imputation within logical sets is difficult. However, it is in the case of few replicates that the presence of missing values, and how they are imputed, can have the most profound impact on the outcome of downstream analyses (e.g. significance analysis and clustering). This study explores the feasibility of imputation across logical sets, using the vast amount of publicly available microarray data to improve imputation reliability in the small sample size setting.

**Results:**

We download all cDNA microarray data of *Saccharomyces cerevisiae, Arabidopsis thaliana*, and *Caenorhabditis elegans *from the Stanford Microarray Database. Through cross-validation and simulation, we find that, for all three species, our proposed imputation using data from public databases is far superior to imputation within a logical set, sometimes to an astonishing degree. Furthermore, the imputation root mean square error for significant genes is generally a lot less than that of non-significant ones.

**Conclusion:**

Since downstream analysis of significant genes, such as clustering and network analysis, can be very sensitive to small perturbations of estimated gene effects, it is highly recommended that researchers apply reliable data imputation prior to further analysis. Our method can also be applied to cDNA microarray experiments from other species, provided good reference data are available.

## Background

The spotted cDNA microarray technology [1] is a widely used tool for examining gene expression profiles across experimental conditions. It measures the messenger RNA (mRNA) levels of thousands to tens of thousands of genes in the sample. Scientists typically conduct the experiments in logical sets, such as time course profiling of yeast cell cycles [2], or comparing cancer to normal tissues [3]. One feature of these experiments is the small number of replicates (technical and/or biological replicated experiments under the same experimental condition): Three or less are common, and six or more are rare. Despite the vast improvement of the technology in recent years, missing values are still a common feature of spotted array experiments. Missing values arise from e.g. blemishes on the chips; a few percent to more than 50 percent of the values of a chip may be missing. Yet most data analysis procedures require a complete data set. Thus missing values need to be imputed, and numerous imputation algorithms have previously been proposed [4-14]. All these studies used only the numerical data from the microarray experiments for imputation. Two recent studies [15,16] incorporated external biological knowledge to improve the estimate.

We begin by briefly reviewing the cDNA microarray technology, for the purpose of introducing a common notation for the rest of the discussion. The spotted cDNA microarray experiments usually follow the two-dye protocol, where the red channel is the sample under study and the green channel is the reference pool [17]. After the microarray experiments are completed, the data of a logical set of *G *genes examined under *C *experimental conditions are collected in a *G *× *C *matrix, which we will denote by *A*. Each row *g *∈ {1, …, *G*} corresponds to a gene, and each column *c *∈ {1, …, *C*} corresponds to a particular microarray sample. After background subtraction and normalization [18], every entry in *A *is the base-two logarithm of the ratio of the red and green intensities.

If a gene *g *∈ {1, …, *G*} has missing values in some columns *c *∈ {1, …, *C*}, most imputation methods will try to borrow strength from other genes *g' *∈ {1, …, *G*} with "similar" expression profiles to *g*, across replicate experiments or experiments under several conditions. For example, KNNimpute [4] first selects the *K *(usually between 10 and 20) genes from {1, …, *G*} with the shortest Euclidean distances to *g, d*(*g, g'*), where

d(g,g′)=∑c∈{1,…,C}:g(c),g′(c) not missing(g(c)−g′(c))2.
 MathType@MTEF@5@5@+=feaafiart1ev1aaatCvAUfKttLearuWrP9MDH5MBPbIqV92AaeXatLxBI9gBaebbnrfifHhDYfgasaacH8akY=wiFfYdH8Gipec8Eeeu0xXdbba9frFj0=OqFfea0dXdd9vqai=hGuQ8kuc9pgc9s8qqaq=dirpe0xb9q8qiLsFr0=vr0=vr0dc8meaabaqaciaacaGaaeqabaqabeGadaaakeaacqWGKbazcqGGOaakcqWGNbWzcqGGSaalcuWGNbWzgaqbaiabcMcaPiabg2da9maaqafabaGaeiikaGIaem4zaCMaeiikaGIaem4yamMaeiykaKIaeyOeI0Iafm4zaCMbauaacqGGOaakcqWGJbWycqGGPaqkcqGGPaqkdaahaaWcbeqaaiabikdaYaaaaeaacqWGJbWycqGHiiIZcqGG7bWEcqaIXaqmcqGGSaalcqWIVlctcqGGSaalcqWGdbWqcqGG9bqFcqGG6aGocqWGNbWzcqGGOaakcqWGJbWycqGGPaqkcqGGSaalcuWGNbWzgaqbaiabcIcaOiabdogaJjabcMcaPiabbccaGiabb6gaUjabb+gaVjabbsha0jabbccaGiabb2gaTjabbMgaPjabbohaZjabbohaZjabbMgaPjabb6gaUjabbEgaNbqab0GaeyyeIuoakiabc6caUaaa@6951@

That is, the distance between genes *g *and *g' *is calculated in the non-missing dimensions. We denote the set of the *K *genes with minimum Euclidean distances to *g *by *S*(*K*). Then, each of the missing values of gene *g *is estimated by a weighted average of expression values for the *K *similar genes. For each *c *such that *g*(*c*)is missing, we impute by the value g˜
 MathType@MTEF@5@5@+=feaafiart1ev1aaatCvAUfKttLearuWrP9MDH5MBPbIqV92AaeXatLxBI9gBaebbnrfifHhDYfgasaacH8akY=wiFfYdH8Gipec8Eeeu0xXdbba9frFj0=OqFfea0dXdd9vqai=hGuQ8kuc9pgc9s8qqaq=dirpe0xb9q8qiLsFr0=vr0=vr0dc8meaabaqaciaacaGaaeqabaqabeGadaaakeaacuWGNbWzgaacaaaa@2E12@(*c*), where

g˜(c)=∑g′∈S(K)(d(g,g′))−1g′(c)∑g′∈S(K)(d(g,g′))−1.
 MathType@MTEF@5@5@+=feaafiart1ev1aaatCvAUfKttLearuWrP9MDH5MBPbIqV92AaeXatLxBI9gBaebbnrfifHhDYfgasaacH8akY=wiFfYdH8Gipec8Eeeu0xXdbba9frFj0=OqFfea0dXdd9vqai=hGuQ8kuc9pgc9s8qqaq=dirpe0xb9q8qiLsFr0=vr0=vr0dc8meaabaqaciaacaGaaeqabaqabeGadaaakeaacuWGNbWzgaacaiabcIcaOiabdogaJjabcMcaPiabg2da9maalaaabaWaaabeaeaacqGGOaakcqWGKbazcqGGOaakcqWGNbWzcqGGSaalcuWGNbWzgaqbaiabcMcaPiabcMcaPmaaCaaaleqabaGaeyOeI0IaeGymaedaaOGafm4zaCMbauaacqGGOaakcqWGJbWycqGGPaqkaSqaaiqbdEgaNzaafaGaeyicI4Saem4uamLaeiikaGIaem4saSKaeiykaKcabeqdcqGHris5aaGcbaWaaabeaeaacqGGOaakcqWGKbazcqGGOaakcqWGNbWzcqGGSaalcuWGNbWzgaqbaiabcMcaPiabcMcaPmaaCaaaleqabaGaeyOeI0IaeGymaedaaaqaaiqbdEgaNzaafaGaeyicI4Saem4uamLaeiikaGIaem4saSKaeiykaKcabeqdcqGHris5aaaakiabc6caUaaa@5DCA@

The reciprocal of the Euclidean distance is used to measure the similarity in expression profiles. Most imputation algorithms are variations of this scheme; they differ by how many genes in {1, …, *G*} are used to impute, and how the weights are calculated.

In this study, we put forth the idea that the matrix *A *may not be the most suitable to impute its own missing values. Intuitively, imputation is best done with a lot of replicates. Yet the logical set is an aggregate of samples under various conditions, with few replicates for each condition. The performance of the imputation method will very much depend on the similarity of the set of genes g' to g. In the case of small logical sets, the limited replicate information or experimental profile information may not be sufficient to determine which genes *g' *are indeed similar to *g*. Thus, this study explores the possibility of using microarrays from different logical sets for imputation. We compare each column *c *in the matrix *A *to hundreds of experiments in databases in the public domain, and we seek experiments with similar expression profiles across the genes to *c*. We will use this richer data source to improve on the identification of genes *g' *that are similar to gene *g *with a missing value in *c*, with the aim of improving imputation accuracy. We demonstrate this meta-data based imputation method using *Saccharomyces cerevisiae *(yeast), *Caenorhabditis elegans *(worm), and *Arabidopsis thaliana *(plant) as the model systems.

To proceed, we need to obtain data from a large number of microarray experiments. This is facilitated by microarray data depositories offering public access, such as the Stanford Microarray Database (SMD) [19]. We downloaded the data of more than two thousand microarrays from SMD, and after some pre-processing, extracted a *database matrix *for each species. The data to be imputed are in the matrix *A*, and its columns will be imputed one by one using "similar" columns in the database matrix. This is where our approach differs from the usual imputation paradigm, under which the matrix *A *is used to impute itself. For computational efficiency, we need to select a subset of the columns from the database matrix to impute a column *c *of *A*. We use the absolute value of column-wise Pearson correlation as a measure of similarity between data columns. Through simulations, we find that imputation via 40 database columns with the highest similarity to the column *c *strikes a good balance between computation efficiency and imputation accuracy, which is measured by the normalized root mean squared error (RMSE).

Our results (1) support the use of the absolute value of column-wise Pearson correlation as a measure of similarity, (2) support the choice of using 40 database columns for imputation, and (3) demonstrate the superiority of our meta-data based approach (imputation via the database matrix) to the usual paradigm (imputation via the matrix *A *itself). Furthermore, for each column *c *in the matrix *A*, we designate the most up-regulated ten percent genes and the most down-regulated ten percent genes as potentially significant. The RMSE of these genes is generally a lot less than that of non-significant ones. In addition, the meta-data imputation greatly improves RMSE for significant genes compared to the usual paradigm. Researchers often use only this filtered subset of genes for clustering and classification, and small perturbations of the estimated gene effects can have a huge impact on these downstream analyses [11]. Thus the database imputation provides high quality data for subsequent analyses. The findings in this study are incorporated in a web-based software tool for yeast, worm, and plant cDNA microarray data imputation [20].

## Methods

Let the microarray data be represented by a matrix, where the *G *rows correspond to the genes and the *C *columns correspond to the samples. We previously described GMCimpute [10]. Briefly, the rows of the matrix are clustered into 1, 2, ..., *Q*-component Gaussian mixtures (*Q *is usually less than 10). For each *q*-component model, we assume that the expression data are generated from a mixture distribution

∑j=1qπjN(μj,Σj),
 MathType@MTEF@5@5@+=feaafiart1ev1aaatCvAUfKttLearuWrP9MDH5MBPbIqV92AaeXatLxBI9gBaebbnrfifHhDYfgasaacH8akY=wiFfYdH8Gipec8Eeeu0xXdbba9frFj0=OqFfea0dXdd9vqai=hGuQ8kuc9pgc9s8qqaq=dirpe0xb9q8qiLsFr0=vr0=vr0dc8meaabaqaciaacaGaaeqabaqabeGadaaakeaadaaeWbqaaGGaciab=b8aWnaaBaaaleaacqWGQbGAaeqaaOGaemOta4KaeiikaGIae8hVd02aaSbaaSqaaiabdQgaQbqabaGccqGGSaalcqqHJoWudaWgaaWcbaGaemOAaOgabeaakiabcMcaPaWcbaGaemOAaOMaeyypa0JaeGymaedabaGaemyCaehaniabggHiLdGccqGGSaalaaa@41FF@

where *π*_*j *_is the mixing proportion, *μ*_*j *_= *μ*_*j*_(1), …, *μ*_*j*_(*C*) is the *j*-th component mean expression profile across the *C *columns, and the *C *× *C *covariance matrix Σ_*j *_summarizes the relationship among the *C *columns. The mixture models are fit to the data by the Classification Expectation-Maximization algorithm (CEM) [21]; then the missing values are estimated by the Expectation-Maximization algorithm [22]; for each missing value, the estimate by GMCimpute is the simple average of the *Q *estimates. If the CEM algorithm takes *I *iterations to converge, then GMCimpute takes *O*(*IQmn*) time. If gene *g *has a missing value in column *c*, we use the information in the other columns {*g*(*c'*), *c' *≠ *c*}, and the estimated relationship among the columns, to impute the value g˜
 MathType@MTEF@5@5@+=feaafiart1ev1aaatCvAUfKttLearuWrP9MDH5MBPbIqV92AaeXatLxBI9gBaebbnrfifHhDYfgasaacH8akY=wiFfYdH8Gipec8Eeeu0xXdbba9frFj0=OqFfea0dXdd9vqai=hGuQ8kuc9pgc9s8qqaq=dirpe0xb9q8qiLsFr0=vr0=vr0dc8meaabaqaciaacaGaaeqabaqabeGadaaakeaacuWGNbWzgaacaaaa@2E12@(*c*) via a weighted average of the component-wise conditional expectations of *g*(*c*)|{*g*(*c'*), *c' *≠ *c*}. That is,

g˜(c)=1Q∑q=1Q(∑j=1qηgjq[μj(c)+Σj[c,−c]Σj[−c,−c]−1(g(−c)−μj(−c))T]),
 MathType@MTEF@5@5@+=feaafiart1ev1aaatCvAUfKttLearuWrP9MDH5MBPbIqV92AaeXatLxBI9gBaebbnrfifHhDYfgasaacH8akY=wiFfYdH8Gipec8Eeeu0xXdbba9frFj0=OqFfea0dXdd9vqai=hGuQ8kuc9pgc9s8qqaq=dirpe0xb9q8qiLsFr0=vr0=vr0dc8meaabaqaciaacaGaaeqabaqabeGadaaakeaacuWGNbWzgaacaiabcIcaOiabdogaJjabcMcaPiabg2da9maalaaabaGaeGymaedabaGaemyuaefaamaaqahabaWaaeWaaeaadaaeWbqaaGGaciab=D7aOnaaDaaaleaacqWGNbWzcqWGQbGAaeaacqWGXbqCaaGcdaWadaqaaiab=X7aTnaaBaaaleaacqWGQbGAaeqaaOGaeiikaGIaem4yamMaeiykaKIaey4kaSIaeu4Odm1aaSbaaSqaaiabdQgaQbqabaGccqGGBbWwcqWGJbWycqGGSaalcqGHsislcqWGJbWycqGGDbqxcqqHJoWudaWgaaWcbaGaemOAaOgabeaakiabcUfaBjabgkHiTiabdogaJjabcYcaSiabgkHiTiabdogaJjabc2faDnaaCaaaleqabaGaeyOeI0IaeGymaedaaOGaeiikaGIaem4zaCMaeiikaGIaeyOeI0Iaem4yamMaeiykaKIaeyOeI0Iae8hVd02aaSbaaSqaaiabdQgaQbqabaGccqGGOaakcqGHsislcqWGJbWycqGGPaqkcqGGPaqkdaahaaWcbeqaaiabdsfaubaaaOGaay5waiaaw2faaaWcbaGaemOAaOMaeyypa0JaeGymaedabaGaemyCaehaniabggHiLdaakiaawIcacaGLPaaaaSqaaiabdghaXjabg2da9iabigdaXaqaaiabdgfarbqdcqGHris5aOGaeiilaWcaaa@7B34@

where ηgjq
 MathType@MTEF@5@5@+=feaafiart1ev1aaatCvAUfKttLearuWrP9MDH5MBPbIqV92AaeXatLxBI9gBaebbnrfifHhDYfgasaacH8akY=wiFfYdH8Gipec8Eeeu0xXdbba9frFj0=OqFfea0dXdd9vqai=hGuQ8kuc9pgc9s8qqaq=dirpe0xb9q8qiLsFr0=vr0=vr0dc8meaabaqaciaacaGaaeqabaqabeGadaaakeaaiiGacqWF3oaAdaqhaaWcbaGaem4zaCMaemOAaOgabaGaemyCaehaaaaa@32AB@ refers to the posterior probability of gene *g *with respect to component *j *in the (*q*-component mixture model, Σ_*j*_[*c*,-*c*] refers to the *c*-th row, and all but the *c*-th column of the covariance of component *j*, and similarly for all other entries.

Let *A' *be the imputed data matrix, i.e., an estimate of *A*. The accuracy of *A' *is measured by normalized root mean squared error (RMSE):

RMSE=mean{(A−A′)2}mean{A2},     (1)
 MathType@MTEF@5@5@+=feaafiart1ev1aaatCvAUfKttLearuWrP9MDH5MBPbIqV92AaeXatLxBI9gBaebbnrfifHhDYfgasaacH8akY=wiFfYdH8Gipec8Eeeu0xXdbba9frFj0=OqFfea0dXdd9vqai=hGuQ8kuc9pgc9s8qqaq=dirpe0xb9q8qiLsFr0=vr0=vr0dc8meaabaqaciaacaGaaeqabaqabeGadaaakeaacqqGsbGucqqGnbqtcqqGtbWucqqGfbqrcqGH9aqpdaGcaaqaamaalaaabaGaeeyBa0MaeeyzauMaeeyyaeMaeeOBa4Maei4EaSNaeiikaGIaemyqaeKaeyOeI0IafmyqaeKbauaacqGGPaqkdaahaaWcbeqaaiabikdaYaaakiabc2ha9bqaaiabb2gaTjabbwgaLjabbggaHjabb6gaUjabcUha7jabdgeabnaaCaaaleqabaGaeGOmaidaaOGaeiyFa0haaiabcYcaSaWcbeaakiaaxMaacaWLjaWaaeWaaeaacqaIXaqmaiaawIcacaGLPaaaaaa@4FE8@

where *A*^2^, for example, is component-wise. We have found that GMCimpute is competitive in terms of imputation RMSE, and in terms of its effect on downstream significance and clustering analysis, and it is also computationally efficient [11]. Traditionally, *A' *is computed solely from *A*. In our meta-data imputation, when we apply GMCimpute to the missing values in the matrix *A*, the columns that are used by GMCimpute are not necessarily limited to only those of *A*. Let us assume that we impute missing values in a column *c *∈ {1...*C*} from matrix *A*. We will identify the *M *columns, from *A *or from the *database matrix D*, with the largest absolute Pearson correlation to column *c*. We will then use these *M *columns in GMCimpute.

In July 2006, we downloaded from SMD [19] the data of 1,082, 469, and 630 cDNA microarrays for yeast, worm, and plant, respectively. Raw data processing (background subtraction and normalization) was performed by SMD. Each entry in the data is the base-two logarithm of the ratio of the red and green intensities. If an experiment used the dye-swap design [17], the two channels were swapped back so that the numerators of the ratios were always the samples under study. The data deposited in SMD over the years came from different microarray platforms. Thus we need to establish the correspondence of genes across the platforms. Yeast has 6,300 or so nuclear open reading frames (ORF), and they are uniquely identified by their ORF systematic names promulgated by the Saccharomyces Genome Database [23]. For the worm, the genes are identified by the clone identifiers maintained by the WormBase [24]. For the plant, the genes are identified by their GenBank Accession Numbers. The data we downloaded from SMD will be used to construct the database matrix *D *(one for each organism).

To examine the performance of meta-data imputation, we need a gold-standard data set on which to conduct a simulation study. We will thus create a data set without missing values. We will then randomly mask values in the gold-standard data set, pretend that they are missing, and apply imputation. Since the true values of these artificial missing entries are known, we can compare and validate imputation methods. For the yeast data, we use yeast ORF systematic names as the row labels, and obtain a 6314 × 1082 matrix. Some entries in this matrix are flagged by the experimenters as missing, and we have to remove rows and columns with too many missing values. After their removal, we obtain a 6220 × 442 matrix. This matrix still has 107,093 missing values (3.9%), and we use the following steps to impute them; (1) We order the columns by the numbers of missing values in them, from the smallest to the largest (i.e. from the easiest to the most difficult to impute); (2) For each column *c *in the order prescribed in Step 1, we identify the 40 columns (*c *excluded) that have the highest absolute values of Pearson correlation to *c*; (3) We impute missing values in *c *using these 40 columns by GMC impute; (4) We repeat Steps 2 and 3 ten times (to reach convergence, as measured by the RMSE between consecutive iterations). Finally, the 6220 × 442 matrix with 3.9% imputed values is the database matrix *D *for yeast. When Steps 2 and 3 are iterated, the imputed values in a column *c*_*i *_may be used to impute the missing values in a different column *c*_*j*_. The reason why we order the columns by the way described in Step 1 is to control the propagation of imputation errors presumably from the smallest to the largest. The imputed values are changing from one iteration to the next, because they are used to impute one another. Thus we need to iterate Steps 2 and 3 some number of times till the change becomes small enough, presumably reaching a (local) optimum. The worm and plant database matrices are similarly prepared. The worm database matrix is 13338 × 381, with 2% imputed values; the plant database matrix is 7424 × 301, with 1.9% imputed values.

In practice, researchers may submit their data for imputation without removing columns or rows with many missing values. However, we recommend a basic quality-control screening. If a sample (microarray) has an excessive number of missing values, it may be better to eliminate it from the logical set.

## Results

We first use the yeast data to develop the algorithm and fine-tune the algorithmic parameters; the parameters are the number of mixtures in GMCimpute and the number of columns from the database matrix *D *selected for use in GMCimpute. Then we use yeast, worm, and plant data to compare imputation via database matrix *D *to imputation within logical sets.

### Measuring Similarity by the Absolute Value of Pearson Correlation

**Figure 1:**
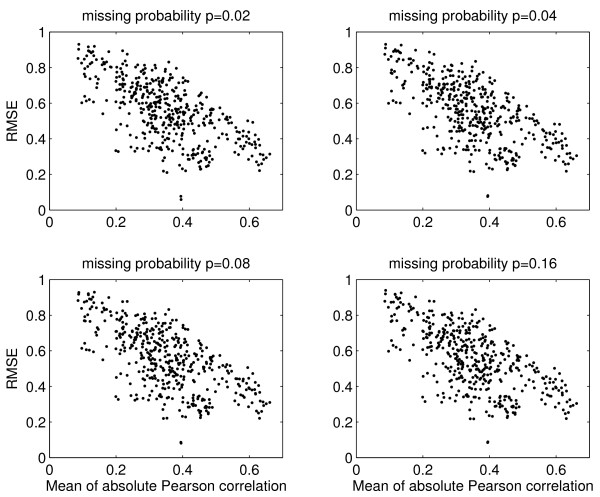
Yeast data: Cross-validation of imputation RMSE of the database matrix. Each point corresponds to a column *c *of the matrix *D*, imputed by using 40 other columns from *D *that have the largest absolute values of Pearson correlation to *c*. The horizontal axis is the mean of absolute Pearson correlation of the 40 columns, and the vertical axis is mean RMSE of 30 independent runs.

**Figure 2:**
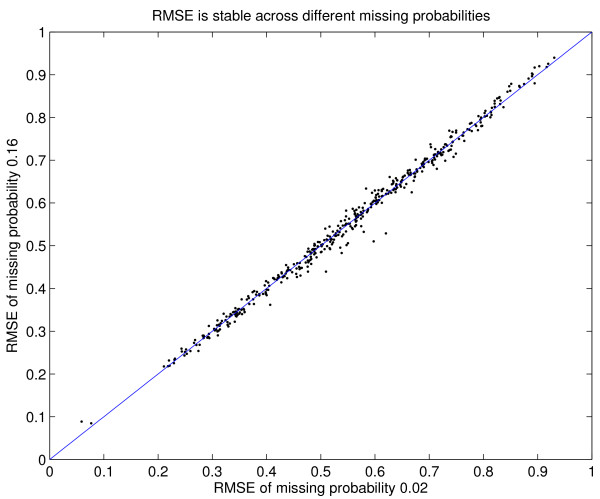
A different view of the data in Figure 1, showing that the imputation RMSEs are stable for different proportions of missing values: Each point corresponds to a column. The (blue) line is *x *= *y*. The horizontal axis is the mean RMSE for 2% missing values, and the vertical axis is mean RMSE for 16% missing values.

We use cross-validation to investigate the imputation accuracy of database columns selected by the absolute values of their Pearson correlation to the column to be imputed. For each column *c *of the yeast database matrix *D*, we randomly generate 2%, 4%, 8%, and 16% missing values in *c *(we prevent these artificial missing values from coinciding with the genuine missing values as flagged in SMD, since this would not constitute a proper validation). Thus the true values of the artificial missing values are known. Then for each instance of *c *with artificial missing values, we select the 40 columns (c excluded) that have the largest absolute values of Pearson correlation to *c*. The missing values are then imputed by GMCimpute of 1, 2, and 3 clusters via the 40 selected columns. The simulation is repeated 30 times for each of the four proportions of missing values. The mean RMSE is plotted in Figure 1. The mean absolute Pearson correlation and the imputation RMSE are highly negatively correlated. This suggests that imputation via the database matrix is a viable approach, as long as there are sufficiently many columns with large absolute Pearson correlation to *c*. Moreover, this result is stable across the full range of 2% to 16% missing values, as shown in Figure 2 and Table 1. Figure 2 shows that the RMSEs for 2% and 16% missing values are highly correlated, and Table 1 contains the Pearson correlation coefficients of the RMSEs for the four proportions of missing values.

**Table 1:** Pearson correlation coefficients of RMSE among the four missing probabilities.

missing prob.	0.02	0.04	0.08	0.16
0.02	1	0.9967	0.9956	0.9961
0.04		1	0.9972	0.9978
0.08			1	0.9989
0.16				1

For imputation within a logical set *A*, the performance of imputation decreases as the proportion of missing values increases. This is well documented in the literature by many authors and us. The reason is simple. When imputing a column *c *from *A*, both *c *and the rest of *A *have missing values. Meta-data imputation is different. It uses information from the *database matrix*, in addition to the data matrix. The columns from the database matrix are complete, independent from the amount of missing values in *c*. Figure 2 and Table 1 show that the performance of meta-data imputation barely decreases with 2% to 16% missing values.

### Number of Columns from the Database Matrix

**Figure 3:**
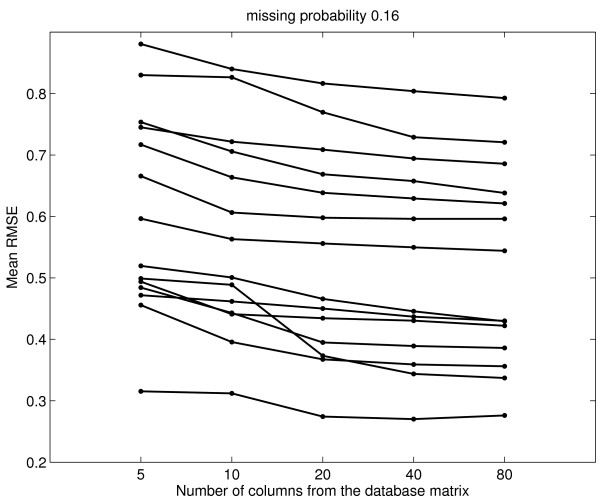
Yeast data: Imputation RMSE when 5, 10, 20, 40, and 80 database columns with the largest absolute Pearson correlation to the column *c *are used to impute *c *with 16% missing values. The results of 15 randomly chosen *c*'s are shown in the figure.

If few database columns exhibit strong correlation to *c*, imputation RMSE is large. Thus one might think that there is a cut-off for the optimum number of database columns to be used for imputation, and that RMSE performance may deteriorate if weakly correlated database columns are included in imputation. We use simulation with increasing numbers of database columns to look for such a cut-off, but do not find one before reaching the limitation of GMCimpute. The limitation is that it becomes difficult to reliably estimate the cluster variances when the data dimension (the number of columns) increases while the number of clusters and thus the numbers of genes in the clusters are held constant. We find that, regardless of strong or weak correlation, the more database columns that are used, the lower the RMSE, as long as the Gaussian mixtures can still be computed. Specifically, we generate 2%, 4%, 8%, and 16% missing values in *c*, and then use 5, 10, 20, 40, and 80 yeast database columns with the largest absolute Pearson correlation to *c *to impute them. The mean RMSE (16% missing values) of 30 independent runs are plotted in Figure 3 for 15 randomly chosen *c*'s. The plots for the other proportions of missing values exhibit the same characteristics. For almost every *c*, RMSE always decreases when the number of database columns increases, although the decrease from 40 to 80 columns is small. In light of these results, we choose to use 40 database columns for imputation as a reasonable trade-off between imputation accuracy and computation efficiency.

### Comparing Imputation via the Database Matrix to Imputation within Logical Sets

**Figure 4:**
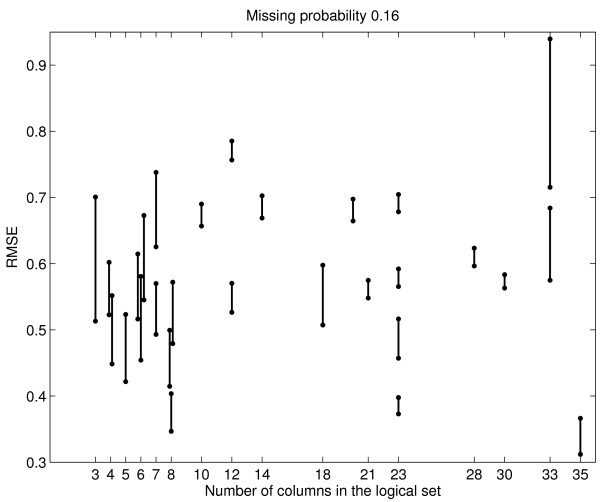
Yeast data: Imputation via the database is always better than imputation within logical sets. Each vertical line represents a logical set; the top of the line is mean RMSE of imputation within the set, and the bottom of the line is mean RMSE of imputation via the database. Some logical sets have the same numbers of columns (the horizontal axis), and their lines are drawn with slight offsets so that they do not overlap.

The most critical issue is whether imputation via the database matrix is better than imputation within a logical set. The 442 columns of the yeast database matrix are partitioned into 28 logical sets, based on their SMD annotation. As an example, one of the logical sets is the time course profile of yeast cells treated with 0.24 mM H_2_O_2_, consisting of 23 columns labeled by 0 to 275 minutes after treatment. The largest logical set has 35 columns, and the smallest has three. As before, 2%, 4%, 8%, and 16% missing values are randomly generated for each of the columns, and then each column *c *is imputed via 40 other database columns. The simulation is repeated 30 times. In each independent run, we also impute *c *via its own logical set, and thus obtain paired comparison of imputation accuracy between meta-data imputation and logical-set imputation. We find that meta-data imputation is always superior to logical-set imputation. The results for 16% missing values are plotted in Figure 4. In particular, the set of H_2_O_2 _treatment has 0.68 RMSE by meta-data, compared to 0.7 RMSE by logical-set. This is one of the smallest improvements among the 28 logical sets, because time course data are highly correlated and thus the two imputation approaches use almost the identical core set of columns. For one of the logical sets with 33 columns, imputation within itself has 0.94 RMSE, and imputation with the database has 0.72 RMSE, an astonishing improvement. Note that meta-data imputation improves imputation accuracy for both large and small logical sets, and that in general the improvement is substantial for experiments with few replicates.

### Comparing Imputation via the Database Matrix to Imputation via One External Logical Set

**Figure 5:**
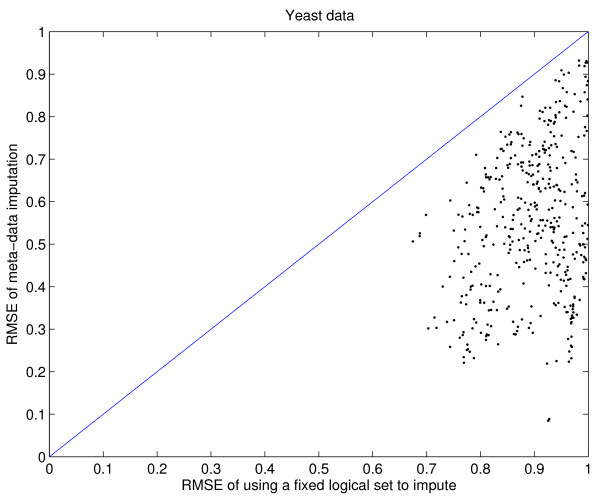
Yeast data: Imputation via the database matrix is always better than imputation via one external logical set. Each point is a column from the yeast database matrix. The horizontal axis is the RMSE of imputation via the H_2_O_2 _set, which has 23 columns, and the vertical axis is the RMSE of imputation via the 23 columns selected from the database matrix using our method. The (blue) line is *x *= *y*. All points are below the line, indicating that the database approach is always superior.

A recent paper [25] also examined imputation via external logical sets. The main finding is that the performance of the Local Least Squares imputation [12] can be consistently improved when extra data, along with the rest of the original data where the sample came from, are included in imputation. Five logical sets are employed in their study. There are 51 samples (columns) in total; the largest set has 18 samples, and the smallest has four. Unlike our approach, which completely breaks up the boundaries of logical sets, their approach either includes or excludes a logical set in entirety. With their approach in mind, we next investigate whether there is any benefit in keeping a logical set together in our setting. We use the time-series of H_2_O_2 _treatment, which has 23 columns, as the reference set. Among the remaining 419 columns of the yeast database matrix, some of them, such as the 2 mM MD treatment, the heat shock treatment, and the hypo-osmotic shock treatment, are parts of the same study of yeast environmental stress response as the H_2_O_2 _set [26]. We assume that they should benefit from the information in the later. In the simulation, for each of the remaining 419 columns, we generate 16% missing values, and impute them via the 23 H_2_O_2 _columns as well as via the 23 columns selected from the database matrix using our method. As shown in Figure 5, breaking up the boundaries of logical sets is always better than using a logical set in entirety in our setting. Furthermore, it is not a trivial exercise to identify the logical sets that lead to good imputation. In contrast, our approach is a simple and very effective method that identifies individual samples from the database for imputation.

### RMSE of Significant and Non-significant Genes

In significance analysis of differential expression [27], statistical procedures are applied to identify genes that are consistently up- or down-regulated across the microarray replicates. Among the thousands of genes in a microarray, only a small portion is declared significant. For imputation to be a useful tool, its impact on downstream analyses should be minimized. Since available analysis tools commonly require a full data matrix, imputed data are usually treated as if truly observed. When imputation is done within a logical set, or, even more extremely, within a gene as in the case of row-mean imputation, this can be quite hazardous as the gene-specific variances are underestimated and can lead to many false positives in the list of significant genes. In other downstream analyses such as clustering, only the subset of genes declared significant are examined. Many of these clustering approaches are sensitive to small perturbations of the estimated gene effects. Thus, imputation accuracy is especially important for the potentially significant genes. In [11] we discussed how imputation can affect significance analyses; in [10] we discussed the impact on clustering.

In this study, we designate for each column the most up-regulated ten percent genes and the most down-regulated ten percent genes as *potentially significant*, and calculate one RMSE for these genes and another RMSE for the non-significant ones. As before, 2%, 4%, 8%, and 16% missing values are randomly generated for each of the columns, and then each column is imputed via 40 other database columns. The simulation is repeated for 30 times.

**Figure 6:**
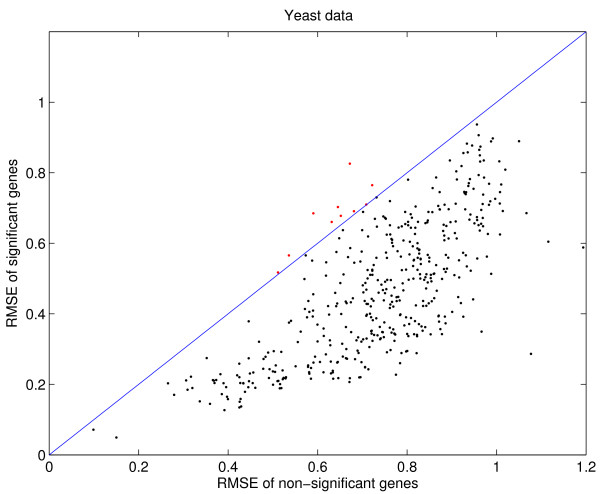
Yeast data: RMSE of significant and non-significant genes. The most up-regulated ten percent genes and the most down-regulated ten percent genes in a column are designated as significant. With 16% missing values imputed by the database matrix, the significant genes generally have less RMSE than the non-significant ones. Each point in the figure corresponds to a column from the database matrix. The (blue) line is *x *= *y*; the (black) points below the line are samples where significant genes have less RMSE than non-significant ones; the (red) points above the line are samples where significant genes have more RMSE than non-significant ones.

For the yeast data, the mean RMSEs of significant and non-significant genes are plotted in Figure 6 for the case with 16% missing values. Each point corresponds to a column of the database matrix. The (blue) line is *x *= *y*; the (black) points below the line are samples where significant genes have less RMSE than non-significant ones; the (red) points above the line are samples where significant genes have more RMSE than non-significant ones. We find that the significant genes generally have much smaller RMSE than the non-significant ones.

**Figure 7:**
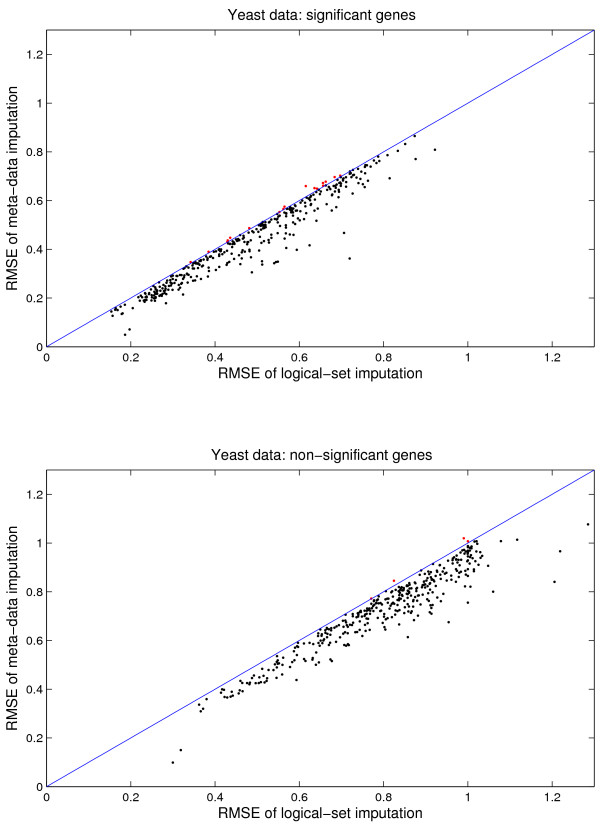
Yeast data: Comparison of imputation RMSE of the meta-data approach and the logical-set approach. With 16% missing values, the meta-data approach generally have less RMSE than the logical-set approach. The legends are the same as Figure 6. The top panel is for significant genes, and the bottom panel is for non-significant ones. This figure offers detailed views of the data presented in Figure 4.

In Figure 7, we compare RMSEs of meta-data imputation to logical-set imputation for the significant genes (the top panel) and the non-significant genes (the bottom panel). This figure offers detailed views of the data presented in Figure 4. Our method improves the imputation accuracy for both significant and non-significant genes with few exceptions. Thus, we can safely deduce that meta-data imputation will have a smaller impact on downstream analyses than the standard approach.

**Figure 8:**
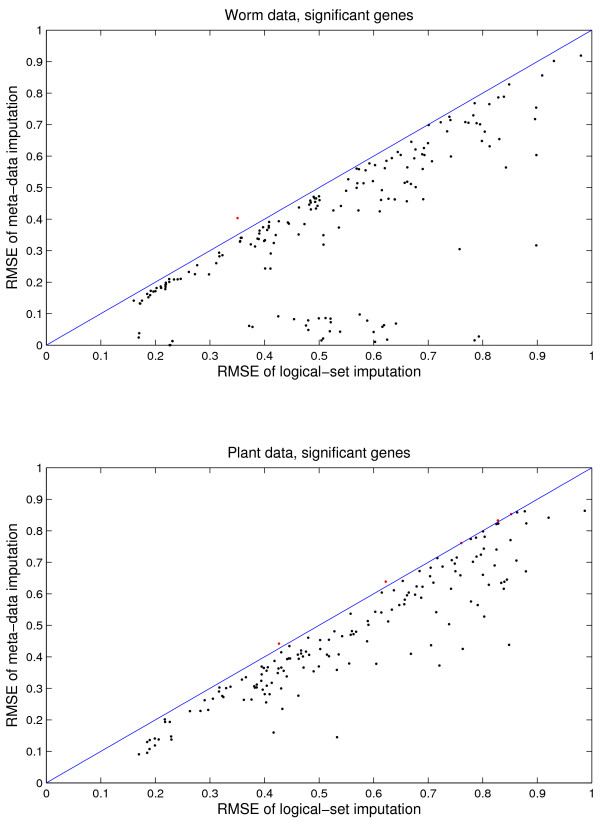
Worm and plant data: Comparison of imputation RMSE of significant genes by the meta-data approach and by the logical-set approach. With 16% missing values, the meta-data approach generally have less RMSE than the logical-set approach. The legends are the same as Figure 6.

For the worm and plant data, all the simulation results are very similar in shapes and trends to the yeast results. Thus we present only the most critical ones, those of the RMSE of significant genes. The top panel of Figure 8 compares RMSE of significant worm genes by meta-data imputation to logical-set imputation, and the bottom panel is for the plant data. In particular, the top panel (worm data) shows that there are a number of columns that have RMSE less than 0.1 by meta-data imputation, compared to RMSE from more than 0.3 up to 0.8 by logical-set imputation.

## Discussion

A logical set of microarray experiments assays mRNA in samples under different conditions, and there are usually few replicates for each condition in the set. Missing value estimation in the literature is mostly confined to imputation within a logical set. However, intuition suggests that imputation would be best done with a lot of replicates. We hypothesize that imputation accuracy for a logical set can be improved by incorporating information from other logical sets of experiments. We download all the *Saccharomyces cerevisiae *(yeast), *Arabidopsis thaliana *(plant), and *Caenorhabditis elegans *(worm) cDNA microarray data from the Stanford Microarray Database [19], and construct database matrices from them. Through rigorous cross-validation and simulation, we validate the new meta-data based imputation with the following results.

First, when a column *c *(data from one microarray experiment) has missing values and when few replicates are available, the next best source of information would be highly correlated columns. Bø *et al*. [8] observed that negative correlation was also helpful in imputation. Thus we use the absolute value of column-wise Pearson correlation to select 40 other columns from the database matrix to impute *c*. Figure 1 shows that absolute Pearson correlation is a useful measure of similarity in that the higher the correlation, the smaller the imputation RMSE. Second, we find imputation via 40 database columns strikes a good balance between computation efficiency and imputation accuracy. Using more columns does improve RMSE, but the improvement diminishes. Third and the most important, we compare logical-set imputation to meta-data imputation. We find that the meta-data approach always performs better, and the superiority can sometimes be astonishing (Figure 4). Fourth, we calculate RMSE for significant and non-significant genes separately, and we find that the former is generally a lot less than the later. That is, the meta-data approach provides smaller RMSE for the important set of potentially significant genes, and thus lessens the impact of imputation on downstream analyses. Sometimes the improvement in RMSE is very dramatic. When combined, these results provide strong support for the application of meta-data imputation before data analysis.

A potential issue in meta-data imputation is the possible presence of lab-specific effects. Typical normalization may not totally remove these effects. To investigate this issue, one would need data from similar experiments conducted in different labs with some within-lab replicates. Then the within-lab and between-lab effects can be properly delineated. At the moment there are not enough data in the public domain to facilitate such an analysis. This is an issue that may further improve imputation.

We construct a simple-to-use web-based tool for meta-data imputation of yeast, worm, and plant cDNA microarray data. Users need to prepare their data in a tab-delimited file format where the first row gives each column a label (such as the experiment name), the first column identifies the rows by unique identifiers, and the rest of the entries are pre-processed and normalized microarray data. The unique identifiers are yeast ORF names, worm clone identifiers in the WormBase, or plant GenBank Accession Numbers. Missing entries are left blank or filled with "NaN" (not a number). The file can be uploaded at the website and imputed data are displayed in the webpage. The computation time is linearly proportional to the numbers of rows and columns, and the number of missing entries. For the yeast with 6,220 ORFs, one column with 1,200 missing entries takes less than 30 seconds to finish, if the load on the server is light.

## Conclusion

The meta-data imputation is a general approach. Its consistently superior performance for yeast, worm, and plant data suggests that it can be applied to other species as well. It is implemented in Matlab scripts. Academic researchers may obtain the scripts by contacting us, and then they can prepare their own database matrices and conduct high quality imputation without transmitting their new data over the internet.

## Authors' contributions

RJ and MO designed the study. MO and HW wrote the Matlab scripts and conducted the simulations. MO constructed the web tool. All authors participated in the preparation of the manuscript and approved its final form.
